# The Technic of Venous Anesthesia1Wiener Medizin. Wochenschrift No. 35, 1909.

**Published:** 1910-05

**Authors:** Fritz Hartel

**Affiliations:** Assistant in the Clinic


### The Technic of Venous Anesthesia[1]

(From the Surgical University Clinics in Berlin. Director: Professor Dr. BIER.)
BY Dr. FRITZ HARTEL, Assistant in the Clinic.
[Translated for BUFFALO MEDICAL JOURNAL.]

THE performance of Bier's venous anesthesia is still pretty generally considered as a difficult and complicated procedure, and it appears that this view is somewhat unfavorable to the wider adoption of the method. Technical consideration might well be regarded as irrelevant where nothing less is at stake than the gaining of a large domain of surgery for the relatively harmless method of local anesthesia; but it must be kept in mind that an unsuccessful local anesthesia which fails to accomplish its object due to imperfect technic, may sometimes cause more harm than a well conducted general anesthesia through psychic injury of the patient and interference with the operation. To conduct a perfectly general anesthesia is by no means easier, however, than the performance of any local anesthesia; but the physician of the present day is apt to underrate the difficulties of an art which he has acquired as the elementary basis of his profession.

The technic of venous anesthesia is simple, and requires nothing but a careful, deliberate carrying out of all the rules, as well as a perfect set of instruments. I take pleasure in obeying the editor's request for another detailed description of this technic, on the basis of 170 cases operated upon in the Royal Clinics.

The indications for the employment of venous anesthesia are easily established. The selection of the operations is governed by this rule: all operations upon the extremities which can be performed under ischemia, and in which simple local anesthesia can not be employed at all, or only imperfectly, belong to the realm of venous anesthesia. Very young children (say those under seven years of age) and individuals with very low intelligence and deficient will power, must be excluded from this as from any other method of local anesthesia. Among general physical conditions, there are only diabetic and senile gangrene to be considered as contraindications, according to our experience. Acute and chronic infectious processes are not a contraindication. Diseased and weakened states of the heart and lungs, instead of being a contraindication, will on the contrary in all cases suggest venous anesthesia even to those operators who otherwise prefer general anesthesia.

The patient is prepared in the usual manner for operation. The affected extremity is completely disinfected and protected with sterile dressings. The following instruments are held in

1. Wiener Medizin. Wochenschrift No. 35, 1909.

readiness for the performance of the anesthesia: a glass receptacle, filled with carbolic acid solution, containing at least three bandages, from 3½ to 6 meters in length, made of thin rubber (the kind of rubber used for constriction-bandages), and provided with strings at the rolled-in end. A graded Janet syringe holding 50 to 100 grams is boiled, rinsed in physiological salt solution, and completely filled with a fresh ½ per cent. sterile novocain solution (0.5 novocain, 0.9 natr. chl. 100.0 aq.), at about body temperature. To the syringe is tied a tube of the best Nélaton rubber, about as thick as a lead pencil. With this tube is connected a canula 1.5 to 2.0 mm. thick and provided with a stopcock; the connection being made through an inserted segment with bayonet closure. The end of the canula is blunt and has a few indented grooves for the fixation of the vein. We also need 1 Pravatz syringe with ½ per cent. novocain solution, 1 scalpel, 2 small three pronged sharp hooks, 3 Deschamps's needles with silk, 1 pair of fine pointed scissors, 2 small hook forceps, a few clamps, ligature threads, needle holders, and a few silk sutures.

The first and indispensable preliminary requirement for the success of the anesthesia is complete ischemia. No satisfaction with venous anesthesia is possible, unless this is carefully attended to. The limb is raised by the assistant, the operator wraps it from the fingers or toes[1] with rubber bandage No. 1, pressing the blood out carefully and thoroughly until a short distance above the site of the injection; the expulsion bandage is then temporarily fixed in place. Immediately above it, the permanent Esmarch bandage is applied as tightly as possible in turns, only partly covering each other, so that the result is not a constricting strand, but as wide a constricting surface as possible. Expulsion bandage No. 1 is then removed, as far as the point above which the lower ischemia bandage No. 3 is to be applied. As bandage 1 is being removed, the limb should appear clear white in color; if it is still red or bluish, the prospects are poor for a satisfactory anesthesia. While the expulsion bandage is still in place at the peripheral end, the lower Esmarch bandage 3 is applied in a similar fashion as bandage 2, and the expulsion bandage is entirely removed. The entire procedure is more easily accomplished, in fact, than described in words.

There are now two constricting bandages around the ischemic extremity. The principle of venous anesthesia consists in filling the segment between the two bandages with a novocain solution, by way of a skin vein, and in this manner to bring all the nerve terminals situated between the bandages in contact with the novo-

1. In septic processes, the expulsion is begun above the infected area.

cain solution, by the natural route of the vascular supply, constituting direct anesthesia. In the second place, all the nerve trunks passing through this region toward deeper parts of the limb are in the same way made incapable of conduction—indirect anesthesia. The space between the two bandages should not be narrower than about 10 cm. and not wider than about 25 cm. (1 to 3 hand's width). In the case of peripheral segments of limbs, direct anesthesia may be carried out under application of a single bandage, but this should never be placed higher than the middle of the forearm, or lower leg.

It is dangerous to select other veins for the injection than the well-known large subcutaneous veins, which should be looked for according to their topographical relations. Often enough, a large blue vein will shimmer most temptingly through the skin at some other point, turning out to be nothing but a skimpy membrane, when exposed under ischemia, which does not permit the introduction of the canula. Hence, the course of the skin veins should first be studied. The position of these vessels is usually described rather superficially, and often inaccurately, in textbooks of anatomy. As a rule, the vein can be felt as a rolling strand under the skin by very gentle palpation; in fat and youthful individuals, the examiner must depend on his topographical knowledge. The large saphenous vein lies at the inner aspect of the thigh, far backward on the adductors, behind the sartorius muscle; at the knee joint, it should be sought for about a thumb's width behind the posterior margin of the femoral condyle; at the leg, the tibial surface underneath the skin should be palpated when the vein will be found lying upon the calf muscles at the mesial margin of this surface; farther downward, it lies in front of the internal malleolus. The small saphenous vein enters more rarely into consideration. The basilic vein of the upper arm lies in the internal bicipital groove and is frequently concealed under the fascia after a short course. It is usually more serviceable than the cephalic vein which lies on the anterior external side of the upper arm, almost in midst of the muscular ridge of the biceps.

The veins of the bend of the elbow and of the forearm take a variable course, and are usually visible under the skin. Only the middle ulnar vein, cephalic vein, or the basilic vein of the forearm should be selected, avoiding all side branches. It should further be kept in mind that the basilic vein is accompanied by the middle cutaneous nerve of the forearm, and the large saphenous vein in the leg is accompanied by the saphenous nerve; these structures should be carefully preserved. In order to be sure to find the vein, it is advisable to study its course prior to the expulsion of blood from the limb, marking the site for the injection of the needle by a needle prick or similar device or possibly by exposing the vein while it is still filled with blood.

Immediately below the upper Esmarch bandage, the transverse skin incision which is to serve for the exposure of the vein is rendered anesthetic for a distance of 2 to 3 cm. by the intra- and subcutaneous injection of ½ per cent. novocain solution; the skin is then divided and is pulled forcibly apart with two sharp hooks by the assistant. As a rule, this causes the vein to appear, but in case it fails to present itself, it is necessary to go deeper into the layer of fat down to the fascia. This illustrates the superiority of the transverse incision which guards against missing the vein by passing on its side into the depth of the tissues. As soon as the vessel appears it is grasped with Deschamps's No. 1 and tied as high as possible at the central end. The sharp hooks are removed and the vein is lifted up with the ligature thread. The vessel is held with Deschamps's 2 under the peripheral end; the thread is left untied, and serves to raise the vein at the other end. The full syringe with the canula, stopcock closed, is lying near the limb ready for introduction. An oblique lateral incision is made with the pointed scissors, in the vein which is stretched out and flattened, until the lumen gapes; the lower corner of the gap is held with the small forceps and the canula is introduced deeply into the lumen, in a peripheral direction. The assistant ties the second thread, while the operator slowly withdraws the canula until the thread slips into the groove: the canula is thus held in place. The assistant holds the canula exactly in the direction of the vein, which is put on the stretch by the central thread; the operator opens the stopcock and slowly injects the fluid, sometimes under considerable pressure. In case some of the solution oozes out from small vessels in the wound which have been divided, these should at once be caught in clamps. If the injection is successful, the superficial veins of the segment will now be seen swelling up and becoming very prominent for a short time, after which the entire segment becomes still whiter in color than heretofore. Sometimes a valve is found to close and oppose a strong resistance against the flow, but this is soon overcome by continuous pressure, and the valve yields. The quantity of solution to be injected amounts to about 80 c.cm. for the leg, and about 50 c.cm. for the arm. A few c.cm. more than necessary should always be injected, in view of the loss incurred by escape of fluid from the wound. The stopcock is now closed, and the tube is removed from the canula, by means of the bayonet closure. The withdrawal of the canula, which is either done at this time, or postponed until the end of the operation, in view of possible further injections, is accomplished in such a way that a Deschamps's 3 is placed under the vein, peripherally from the canula, the vessel is tied, thread 2 is divided, and the canula is withdrawn. The wound is closed by sutures.

What do we require of a successful venous anesthesia?

1. *Direct Anesthesia.*—Immediately, or a few minutes after completion of the injection, the segment of limb between the two bandages is entirely anesthetic, for all sorts of operations. The anesthesia extends to all the tissues, including the nerve trunks passing through the segment. It is only above and close to the Esmarch bandage that there remains a skin zone capable of conduction, its width amounting to a few millimeters on the side of the injected vein, and sometimes up to 2 or 3 cm. at the opposite side. Hence, the bandage should be applied high enough from the start, so that it is not necessary to operate in this region. In case the constricted segment is very large, the onset of the direct anesthesia at the opposite side from the vein may be delayed up to five minutes, but it should not last longer than that. In the case of a small limb, in thin individuals, and in children, the operation can be begun immediately after the injection; when the proportions are extensive, however, it is preferable to wait about five minutes. As the deep parts are the first to become anesthetic, one may start, in the case of joints, with the mobilisation of ankylosis, and so forth.

2. *Indirect Anesthesia.*—It frequently happens that at the termination of the injection, the segment of the limb below the peripheral bandage is also rendered at once either totally or partially anesthetic; but, as a rule, from 5 to 15 minutes will pass until the sensation has become completely paralysed, the anesthesia progressing from the side of the injection toward the opposite side, and from above downward. This sensory paralysis is followed from 0. to 2 minutes later by motor paralysis of the extremity. After a suitable delay, such as we are accustomed to in a similar fashion in the methods of local conduction—anesthsia, the entire bandaged extremity is completely anesthetic, with loss of posture sense, and flaccid muscular paralysis. The lower bandages may now be removed. It is now possible to carry out upon the limb any bloody operation not only, but the muscular relaxation may be utilised to good advantage for the correction of deformities, the replacement of fractures and luxations, and so forth. It was found that the relaxation of the muscle is actually more prompt and extensive under these conditions, than under ether anesthesia.

3. *Duration of the Anesthesia.*—There is no other method in which the duration of the sensory paralysis is so absolutely under our control, as in venous anesthesia. It persists as long as the Esmarch bandage is left in place, and disappears soon after the removal of the latter. It is advisable, however, not to prolong the time of the operation unnecessarily, because it has been noted that while the anesthesia does not vanish under prolonged application of the bandage (our longest operation consumed 72 minutes)—but in certain cases, the pressure of the bandage may become distressing to the patient. These disturbances are, however, entirely out of proportion with the severe pains which appear in local anesthesias, after prolonged application of the Esmarch bandage, and often interfere in a most inconvenient fashion with the operation, for in venous anesthesia, the anesthetic agent which is becoming diffused underneath the bandage, serves to remove the direct pressure pain and no paresthesias can develop in the constricted limb, on account of the total anesthesia. Yet it is desirable that even slight disturbances in venous anesthesia be corrected. Complaints as to the pressure from the bandage were noted by us in 6 per cent. of cases. The duration of the operations, in these cases, usually amounted to more than one hour (tendon-plastics, tendon sutures, and the like), whereas the average duration of our operations under venous anesthesia in general amounted to 37 minutes. This request of a short time for the operation should not be counted against venous anesthesia! Each form of anesthesia requires certain definite precautions. Whereas the old surgeons were obliged to take bodily hold of their patients, and perform their amputations within a few seconds, we have probably become spoiled in regard to the time for the intervention, by general anesthesia; but here also it should be kept in mind that the danger of the anesthesia increases with the length of its employment. Injection anesthesia in its turn, requires a peculiar skill and dexterity on the part of the operator, without restricting the time of the operation.

The second condition of venous anesthesia, addressed to the operator, is this, that the operation must be entirely completed before the bandage is removed. Hemostasis and suture must be performed with the bandage in place. If the bleeding vascular lumina can not be recognised in the empty state, physiological salt solution should be injected through the canula, and this will spurt in a jet from the damaged vessels, so that they can be seized.

4. *End of the Anesthesia.*—After the removal of the bandage, motion and sensation return within a few minutes. The length of the operation has no bearing upon the rapidity of this return. The assumption that the inflowing blood sweeps the poison away mechanically, should be repudiated; for if physiological salt solution be injected by the same route through which the novocain has been introduced, the former will escape in profuse jets from the operative wound, while the anesthesia persists, as we were enabled to convince ourselves in a number of cases. If the bandage is loosened for an instant and then retightened, the anesthesia is likewise preserved or it is diminished to but a slight degree.

The motor paralysis also is not influenced at all by the salt injection, and only partially so by the short interrupted entrance of blood. These observations convincingly confirm the fact that the poisonous anesthetic forms a compound with the cells of the body which these are only enabled to dissolve with the aid of nutrition by arterial blood. The fact that the poison is returned to the circulation under an altered form has been demonstrated by animal experiments and also by the observation that none of our patients presented any signs of intoxication, after the removal of the bandage.[2]

A brief discussion is still in order, as to the behavior of the individual nerve functions and qualities of sensation, in venous anesthesia. The sensory nerves are more promptly paralysed than the motor nerves. Among the qualities of sensation, repeated careful observation has shown the temperature sense to be lost first of all, then the pain sense, and then the tactile sense; these functions returning in the reversed order, in the same manner as has been demonstrated in cocain paralysis.

The addition of suprarenal preparations, to the anesthetic, in order to prolong the duration of the anesthesia beyond the time when the bandage is removed, is not considered by us as advisable, after our last experiments with Braun's tablets. The uniform distribution of the anesthetic in the segment of limb was sometimes impaired by the adrenalin, while a prolongation of the anesthesia was obtained only in a few instances.

The number of operations hitherto performed by us under venous anesthesia amounts to 170. There is no need to enter in this connection into a detailed discussion of the individual operations and the necessary technic. A thorough knowledge of the principal rules will serve for guidance in individual cases which have occasionally disturbed the course of the operation,

2. Except two cases of slight malaise, which are mentioned in Bier's original contribution (*Berliner Klinisch Wochenschrift*) No. 11, 1909: (1) A tendency to vomiting appeared during the operation in a child, after the solution had been injected in a central direction; (2) A tendency to vomiting likewise manifested itself in the case of a very frail woman, fifteen minutes after the operation. The same is occasionally noted in local anesthesia.

or led to a failure of the anesthesia in general. Such cases are as follows:

1. Too low a dosage, in regard to (a) concentration, or (b) quantity of the anesthetic.

2. Disturbances during the injection.

3. Employment of very small atypical veins.

4. Alteration in veins caused by diseased conditions.

5. Imperfect application of the constricting bandage (a) central, and (b) peripheral bandage.

6. Addition of adrenalin.

Under (1)—A concentration below 0.5 per cent. frequently yields incomplete anesthesias. At the present time, we employ exclusively the 0.5 per cent. solution, but the 1 per cent. solution is also very desirable. The quantity of the 0.5 per cent. solution amounts to 40 to 50 c.cm. for the upper extremity, and 70, 80, 100 for the lower extremity.

Under (2)—If the instruments are defective, or the canula has not been introduced properly into the lumen of the vein, a part of the solution will be lost in the same way, and the anesthesia thus becomes imperfect. In the course of time, we have learned to avoid these complications fairly certainly.

Under (3 and 4)—Atypical veins, as stated above, are unreliable, interfering as they do with the introduction of the canula. In a similar way, those veins should be avoided which lie embedded in cicatricial tissue, as they may under certain conditions prevent the occurrence of the anesthesia.

Varicose veins, occupy a place apart in so far as anesthesia here frequently follows only on the inner side. This is sufficient for the operation on the varicose veins themselves; but in other operations, this must be guarded against by a somewhat higher dosage.

Under (5)—If the limb is not thoroughly anemic, the appearance of the anesthesia is considerably delayed. Very loosely applied bandages will permit the solution to escape in an upward or downward direction.

Under (6)—See above.

The disturbances of the anesthesia which arise as the result of these causes are very characteristic. Most frequently they concern the direct anesthesia. Whereas the anesthesia normally is outlined in shape of a dome against the central bandage, omitting the strip of skin near the bandage as stated above, this sentient strand in case of imperfect anesthesia will extend down to the peripheral bandage, on the opposite side. A certain area of the opposite side is not anesthetised at all, or the anesthesia is much belated. It is also understood that the indirect anesthesia may be complete, while the skin on the opposite side of the direct area is still possessed of sensation, as was occasionally noted in our cases.

Disturbances in the direct anesthesia are the most common when the segment between the bandages is too narrow, or when the peripheral bandage is too loosely applied. The anesthetic under these conditions does not take the path of capillary infiltration of the area of direct anesthesia, under compulsion of the constricting bandages, but it diffuses arbitrarily through the vascular channels of the entire extremity.

There are but a few cases in which the cause of failure (in one instance) or imperfect outcome, could not be discovered, and we may state that under careful observance of the technical regulations, and a proper selection of the patients, it is possible to reduce the failures and the incomplete anesthesias, in venous anesthesia, to a small minimum which does not enter into consideration, as compared with the general perfection and far-reaching importance of this method.

